# Strain Modal Analysis of Small and Light Pipes Using Distributed Fibre Bragg Grating Sensors

**DOI:** 10.3390/s16101583

**Published:** 2016-09-25

**Authors:** Jun Huang, Zude Zhou, Lin Zhang, Juntao Chen, Chunqian Ji, Duc Truong Pham

**Affiliations:** 1 School of Mechanical and Electronic Engineering, Wuhan University of Technology, Wuhan 430070, China; zudezhou@whut.edu.cn (Z.Z.); chenjt.0805@163.com (J.C.); 2 School of Automation Science and Electrical Engineering, Beihang University, Beijing 100191, China; johnlin9999@163.com; 3 School of Engineering, University of Birmingham, Birmingham B15 2TT, UK; c.ji@bham.ac.uk (C.J.); d.t.pham@bham.ac.uk (D.T.P.)

**Keywords:** small and light pipe, strain modal analysis, Fibre Bragg Grating, strain mode shape

## Abstract

Vibration fatigue failure is a critical problem of hydraulic pipes under severe working conditions. Strain modal testing of small and light pipes is a good option for dynamic characteristic evaluation, structural health monitoring and damage identification. Unique features such as small size, light weight, and high multiplexing capability enable Fibre Bragg Grating (FBG) sensors to measure structural dynamic responses where sensor size and placement are critical. In this paper, experimental strain modal analysis of pipes using distributed FBG sensors ispresented. Strain modal analysis and parameter identification methods are introduced. Experimental strain modal testing and finite element analysis for a cantilever pipe have been carried out. The analysis results indicate that the natural frequencies and strain mode shapes of the tested pipe acquired by FBG sensors are in good agreement with the results obtained by a reference accelerometer and simulation outputs. The strain modal parameters of a hydraulic pipe were obtained by the proposed strain modal testing method. FBG sensors have been shown to be useful in the experimental strain modal analysis of small and light pipes in mechanical, aeronautic and aerospace applications.

## 1. Introduction

Vibration fatigue of hydraulic pipes subjected to dynamic forces is one of the major failure factors in the mechanical, automotive, and aeronautic industries [1]. Therefore, experimental modal analysis or testing of pipes is essential for understanding and evaluating their dynamic characteristics in design and operation. Electrical strain gauges and accelerometers are commonly used for strain and displacement modal analysis of pipes to consider dynamic properties and fatigue strength [2]. However, these conventional approaches face significant challenges where the size, weight, amount and location of sensing elements are issues, especially for the small and light pipes in the aeronautic and aerospace fields [3,4]. For example, strong electromagnetic interference may affect the results of strain gauges and accelerometers for multipoint and accurate response measurements. Accelerometers installed on the tested pipes would bring a mass-loading effect which would affect the dynamic characteristics of the pipes [5,6].

Unlike conventional electrical techniques, Fibre Bragg Grating (FBG) sensors adopt light as a sensing signal, which gives FBG sensors various attractive characteristics, such as small size, light weight, immunity to electromagnetic interference, and large multiplexing capability [7,8,9]. In recent years, FBG sensors have been investigated and applied for dynamic strain measurement and modal analysis [10,11,12,13]. For example, distributed FBG sensors were used for strain modal analysis and damage identification of a plate structure [14], and embedded fibre grating sensors were utilised in experimental modal analysis on an aircraft wing model [15]. FBG optical sensors were chosen for monitoring the dynamic response of a composite antenna sub-reflector [16]. Strain modal analysis of a helicopter main rotor blade using FBG sensors was reported [17] and modal analysis of a helicopter blade model using a long FBG interrogated by optical frequency domain reflectometry was demonstrated [18].

However, the strain modal measurement of hydraulic pipes has not been studied widely. On the basis of the previous research of the dynamic strain measurement of hydraulic systems [19], FBG sensors are used for experimental strain modal analysis of small and light pipes in this paper. The theoretical backgrounds including the matrix of strain frequency response function and the modal parameter identification method are described. An experimental strain modal testing and analysis case of a cantilever pipe using distributed FBG sensors is carried out. As a reference, the natural frequencies and strain mode shapes are calculated with finite element simulation. The analysis results and modal assurance criteria (MAC) values indicate that the natural frequencies and strain mode shapes of the tested pipe acquired by FBG sensors are in good agreement with the results obtained by the reference accelerometer and simulation outputs. The strain modal parameters of a hydraulic pipe were obtained by the proposed strain modal testing method using FBG sensors. Distributed FBG sensors could be widely used in the experimental strain modal analysis of small and light pipes in mechanical, aeronautic and aerospace applications.

## 2. Theoretical Background

Although the strain mode shape and displacement mode shape are both intrinsic dynamic characteristics of a structure and they correspond to each other, the strain mode shape can capture local structural defects (such as cracks) more effectively [20]. By investigating the stress-strain distribution, experimental strain modal analysis is important for vibration fatigue analysis and damage identification [21]. Their light weight and excellent multiplexing capability enable FBG sensors to capture not only the local key parts of the structure but also the overall dynamic response information in experimental strain modal analysis [22].

### 2.1. Strain Modal Analysis

The frequency response function (FRF) is defined as the ratio of the output response of a structure due to an excitation force, which can be obtained by transforming the measured time data (input load and output response) from the time domain to the frequency domain using the fast Fourier transform (FFT) algorithm. The strain frequency response function (SFRF) is a fundamental measurement that isolates the inherent dynamic properties of a mechanical structure. The excitation force and structural strain responses have to be measured simultaneously to define the SFRFs. Modal parameters (frequency, damping, and strain mode shape) can be obtained from a set of SFRF measurements. The matrix of the SFRF [H^ε^] is given by [20]:

(1) $${\left[H^{\varepsilon }\right]}_{N_{o}N_{i}}=\sum_{r=1}^{m}Y_{r}\left[\begin{array}{cccc} \psi_{1r}^{\varepsilon }\varphi_{1r} & \psi_{1r}^{\varepsilon }\varphi_{2r} & \cdots & \psi_{1r}^{\varepsilon }\varphi_{N_{i}r}\\ \psi_{2r}^{\varepsilon }\varphi_{1r} & \psi_{2r}^{\varepsilon }\varphi_{2r} & \cdots & \psi_{2r}^{\varepsilon }\varphi_{N_{i}r}\\ \cdots & \cdots & & \cdots \\ \psi_{N_{o}r}^{\varepsilon }\varphi_{1r} & \psi_{N_{o}r}^{\varepsilon }\varphi_{2r} & \cdots & \psi_{N_{o}r}^{\varepsilon }\varphi_{N_{i}r} \end{array}\right]$$

(2) $$Y_{r}={\left(k_{r}-\omega^{2}m_{r}+j\omega c_{r}\right)}^{-1}$$

where *N_i_* represents the number of inputs and *N_o_* represents the number of output measurements; *m* is the total number of modes considered; $\psi_{ir}^{\varepsilon }$ is the normalised value of the *r*-th strain mode at point *i*, and *ϕ_lr_* is the normalised value of the *r*-th displacement mode at point *l*; *k_r_*, *m_r_*, and *c_r_* are the *r*-th modal stiffness, modal mass and modal damping, respectively; *ω* is the frequency of excitation. The columns of the matrix correspond to the strain responses due to the excitation points along the rows of the matrix. The elements of the SFRF matrix can be expressed as:

(3) $$H_{il}^{\varepsilon }=\sum_{r=1}^{m}\frac{\psi_{ir}^{\varepsilon }\varphi_{lr}}{k_{r}-\omega^{2}m_{r}+j\omega c_{r}}$$

The element of the SFRF matrix is the ratio between the Fourier transform of the strain response at point *i* induced by an excitation force applied at point *l*. From Equations (2) and (3), it can be found that any column of the SFRF matrix contains the information about the strain mode $\psi_{r}^{\varepsilon }$ for all the modes, any element of the SFRF matrix contains the information about *k_r_*, *m_r_*, and *c_r_* for all the modes. Therefore, it is only necessary to excite one selected point and acquire the strain responses at all the measurement points, which enable the modal parameters (frequency, damping, strain mode shape) to be obtained after data processing.

### 2.2. Strain Modal Parameter Identification

After the SFRFs are acquired by measurement and data processing of strain responses, the next step is the modal parameter identification, which is the process of estimating a parametric model (poles and mode shapes) [16]. There are several methods proposed for modal parameter identification such as the complex exponential algorithm (CEA), least squares complex exponential (LSCE), polyreference frequency domain (PFD), and eigensystem realisation algorithm (ERA).

The SFRF can be described by the residues (in the numerator) and the poles (in the denominator) for each of the modes of the system with *m* DOFs (degrees of freedom).

(4) $$H_{il}^{\varepsilon }\left(s\right)=\sum_{r=1}^{m}\left[\frac{R_{ilr}}{2j\left(s-s_{r}\right)}-\frac{R_{ilr}^{*}}{2j\left(s-s_{r}^{*}\right)}\right]$$

(5) $$R_{ilr}=\frac{\psi_{ir}^{\varepsilon }\varphi_{lr}}{\sqrt{1-\zeta_{r}^{2}}\omega_{r}m_{r}}$$

(6) $$s_{r}=-\zeta_{r}\omega_{r}+j\sqrt{1-\zeta_{r}^{2}}\omega_{r}$$

where *R_ilr_* and *s_r_* are the *r*-th modal residue and pole, respectively; *ω_r_* and *ζ_r_* are the *r*-th undamped natural frequency and damping ratio, respectively. The method of rational fraction orthogonal polynomial (RFOP) is used to estimate the poles of the system in this paper. The poles of the system can be obtained by power polynomial solutions and stabilisation diagrams. The stabilisation criterion about frequency, damping and mode shape can be utilised to acquire the number of the poles.

When the poles are estimated, the method of least-squares frequency-domain (LSFD) was used to estimate the strain mode shape vector from the measured SFRFs. The estimation model of the strain frequency response function is given by:

(7) $${\left[H^{\varepsilon }\left(\omega \right)\right]}_{N_{o}N_{i}}=\sum_{r=1}^{N}\left[\frac{\left[R_{r}\right]}{2j\left(j\omega -s_{r}\right)}-\frac{\left[R_{r}^{*}\right]}{2j\left(j\omega -s_{r}^{*}\right)}\right]-\frac{\left[L\right]}{\omega^{2}}+\left[U\right]$$

where [*L*] is the lower residual matrix, and [*U*] is the upper residual matrix. When the poles of system *s_r_* are applied to Equation (7), the strain frequency response function can be transferred to the linear equation related to residues, upper residuals and lower residuals, which could be solved by the least square method.

As any column of the SFRF matrix contains all the information about the strain modes, according to Equation (4) the residue vector [*R_r_*] of one column can be expressed as:

(8) $$\left[R_{r}\right]={\left[\begin{array}{l} R_{1r}\\ R_{2r}\\ \vdots \\ R_{N_{o}r} \end{array}\right]}^{T}=\frac{\varphi_{lr}}{\sqrt{1-\zeta_{r}^{2}}\omega_{r}m_{r}}\left[\begin{array}{l} \psi_{1r}^{\varepsilon }\\ \psi_{2r}^{\varepsilon }\\ \vdots \\ \psi_{N_{o}r}^{\varepsilon } \end{array}\right]$$

According to Equation (8), the normalised strain mode shape vector can be obtained.

## 3. Experiment and Comparison with Finite Element Analysis (FEA) Simulation

To investigate the strain modal analysis of pipes using distributed FBG sensors, a strain modal testing and analysis case was carried out. The measured modal parameters of natural frequencies and strain mode shapes were compared with the results of the reference accelerometer and finite element analysis (FEA).

### 3.1. Experimental Setup

**Figure 1:**
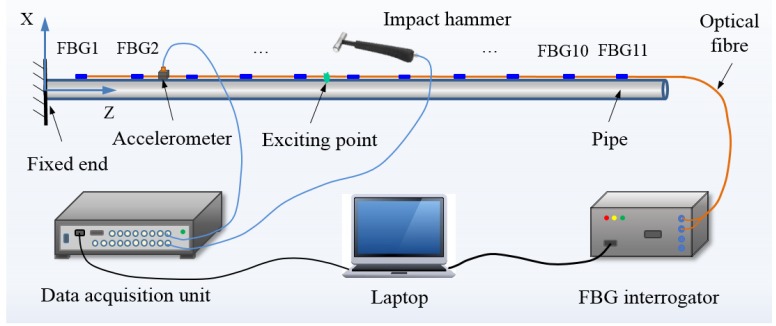
Schematic diagram of experimental setup.

The schematic diagram of the experimental strain modal testing setup is depicted in Figure 1. It mainly contains a small-diameter cantilever pipe, accelerometer, impact hammer, FBG sensors, data acquisition unit, FBG interrogator, laptop, and optical fibre. The impact hammer (2302-10, Endevco, San Juan Capistrano, CA, USA) with a load cell was used to excite the pipe at the exciting point (as shown in Figure 1). An accelerometer (4508B, B & K, Nærum, Denmark) with mass of 4.8 g was bonded on the surface of the pipe as a reference measurement to detect the acceleration response of the tested pipe along the *X* axis direction, as shown in Figure 1. Eleven FBG sensors were installed on the surface of the tested pipe (as shown in Figure 1) by epoxy adhesive to measure strain responses at different points. A data acquisition and signal analysis system (PULSE 7700, B & K,) was used to collect and process the experimental data of the accelerometer and impact hammer. A dynamic FBG interrogator with a maximum sample rate of 4 kHz, a minimum wavelength resolution of 1 pm, and four channels was employed to detect the wavelength shifts of FBG sensors in real time. The dynamic FBG interrogator is based on the tunable Fabry-Perot (F-P) filter technology.

The inner diameter, wall thickness and length of the tested pipe are 13 mm, 1.5 mm, and 985 mm, respectively. One end of the pipe was fixed and the other one is free as shown in Figure 1. The material of the tested pipe is 304 stainless steel and its density, Young’s modulus, and Poisson’s ratio are 7930 kg/m^3^, 200 GPa, and 0.31, respectively.

The FBG sensors were glued on the surface of the tested pipe and spaced in intervals of 90 mm along the length (*Z* direction) to detect the strain responses under impacting load induced by the hammer. The initial wavelengths and locations of the FBG sensors are listed in Table 1. Theses FBG sensors were multiplexed in two optical fibre cables connected to two channels (Ch1: FBG1, FBG2, FBG3, FBG4, FBG5 and FBG6; Ch2: FBG7, FBG8, FBG9, FBG10 and FBG11) of the FBG interrogator.

### 3.2. Finite Element Analysis

**Figure 2:**
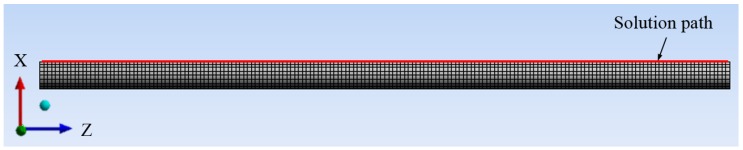
Finite element geometry model.

To validate experimental results, numerical modal analysis of the tested pipe under investigation was performed with finite element analysis using the software ANSYS to determine natural frequencies and normalised strain mode shapes. The finite element geometry model with the mesh of the pipe is developed, as shown in Figure 2. The left end of the pipe is fixed and the other one is free. A coordinate system is created at the fixed end of the pipe. A solution path on the surface of the pipe along the length (*Z* direction) is chosen to acquire the strain responses in different strain mode shapes.

## 4. Results and Discussion

**Figure 3:**
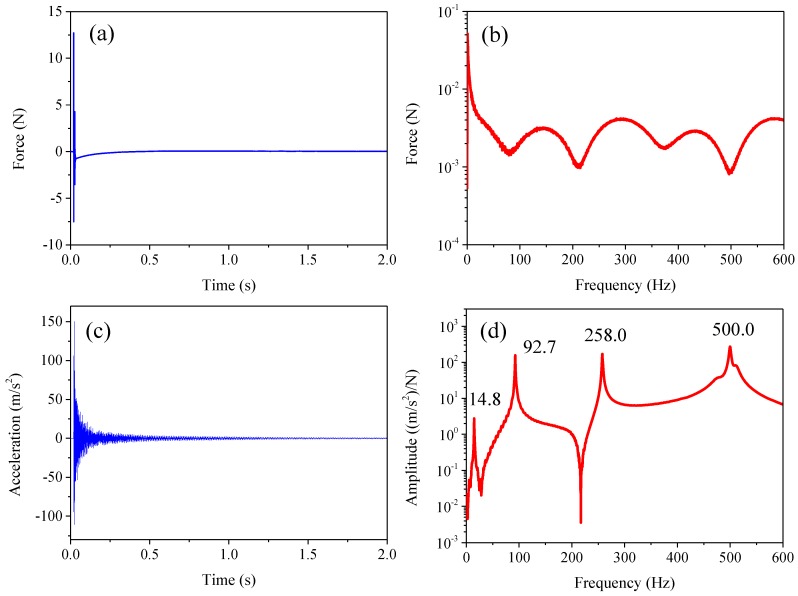
Signals of impacting force and acceleration. (**a**) impacting force; (**b**) frequency spectrum of impacting force; (**c**) acceleration; (**d**) acceleration FRF.

The impacting force and dynamic acceleration response were measured simultaneously as the input excitation and output response of the acceleration FRF. The time domain signals associated with the impact hammer and related acceleration response acquired by the accelerator are shown in Figure 3a,c, respectively. The frequency domain of the impacting force is obtained by using the FFT algorithm, as shown in Figure 3b. Figure 3d shows the estimated acceleration-over-force FRF and the identified first four natural frequencies which occur at 14.8 Hz, 94.7 Hz, 258.0 Hz and 500.0 Hz with a frequency resolution of 0.1 Hz.

**Figure 4:**
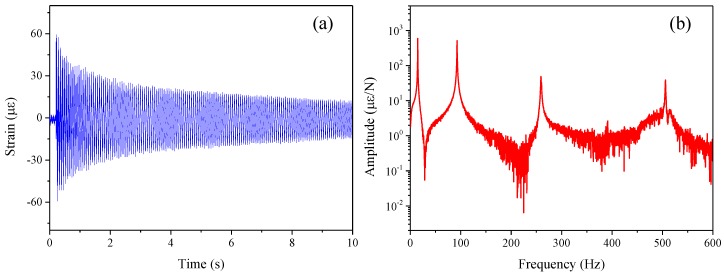
Signal of FBG sensor (FBG1). (**a**) strian response; (**b**) strian frequency response function.

The strain responses of the tested pipe under the impacting load induced by the hammer are captured by the 11 FBG sensors. The time domain signal of one of the FBG sensors (FBG1) is shown in Figure 4a. As the response does not decay to zero before the end of the sampling window, an exponential window is used to reduce the leakage effects in the response spectrum. Using the FFT algorithm, the strain SFRF obtained by FBG1 sensor is shown in Figure 4b. The other SFRFs of the FBG sensors could also be obtained in the same way. Therefore, one column of the SFRF matrix could be acquired for the strain modal parameter identification of the tested pipe.

**Figure 5:**
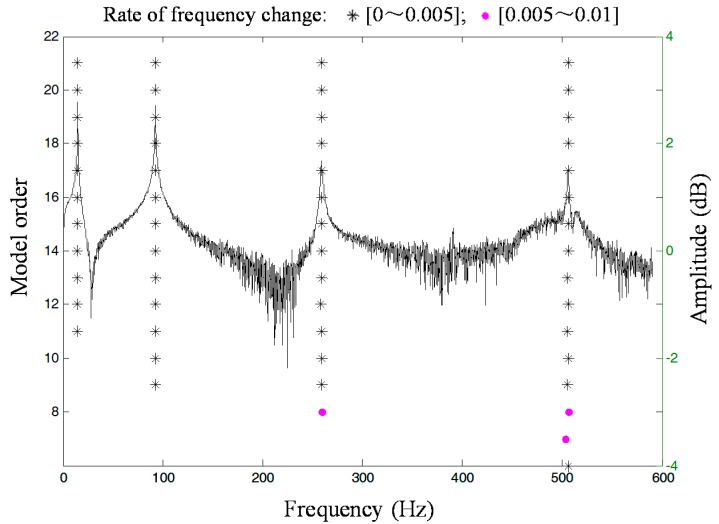
Stabilisation diagram.

The RFOP method mentioned above was applied to estimate the modal parameters of the pipe from a set of SFRFs measured by FBG sensors. According to Equations (4)–(6), the poles of the pipe could be estimated. The stabilisation diagram is represented in Figure 5. As the exponential window adds artificial damping to all of the modes of the pipe in a known manner, the estimated damping ratios after curve fitting could be obtained by subtracting the artificial damping. The estimated first four modal parameters (natural frequencies and damping ratios) of the tested pipe from a set of SFRFs obtained by FBG sensors can be found in Table 2.

**Figure 6:**
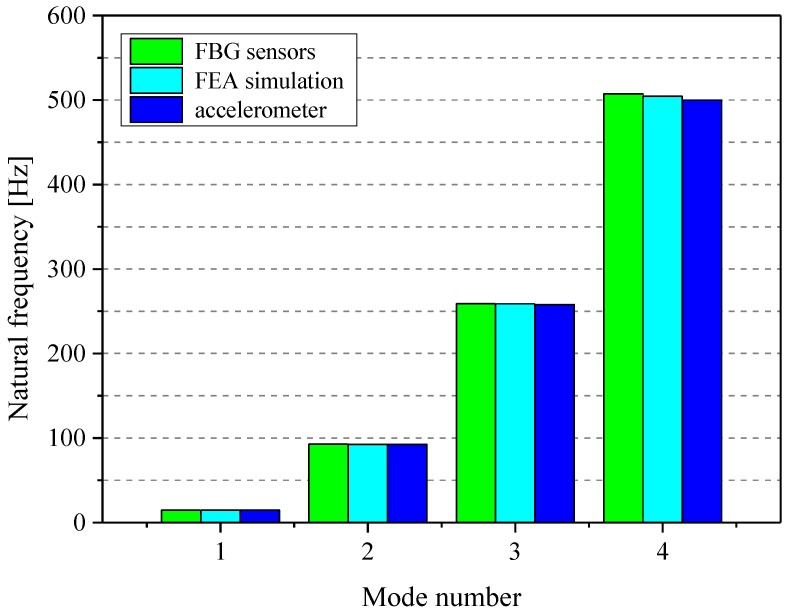
Comparison of experimental and analysis natural frequencies.

The comparisons of natural frequencies from experimental measurements (FBG sensors and accelerometer) and numerical analysis (FEA simulation) are depicted in Figure 6. The experimental results demonstrate a good agreement between the natural frequencies provided by FBG sensors and FEA simulation calculation results.

It is observed that unlike the natural frequencies obtained by FBG sensors, the third and fourth mode natural frequencies captured by the accelerometer are, respectively, 0.35% and 0.93% smaller than the results calculated by FEA simulation. Similarly, the accelerometer-measured third and fourth mode natural frequencies are 0.39% and 1.44% smaller than the results obtained by FBG sensors, respectively. It is mainly caused by the mass of the accelerometer placed on the pipe. The mass added to the pipe by the accelerometer has a negative effect on the modal testing, which causes the measured natural frequencies to be smaller than the correct values.

**Figure 7:**
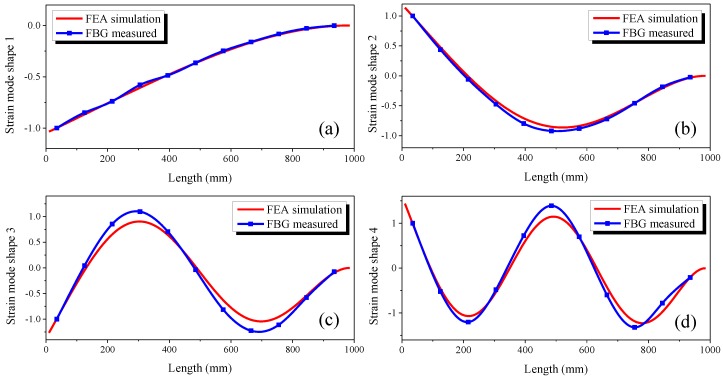
Comparison of normalised strain mode shapes between the results of FEA simulation and experiment, the strain mode shapes along the Z direction of the pipe: (**a**) mode 1; (**b**) mode 2; (**c**) mode 3; (**d**) mode 4.

After the poles of the tested pipe are estimated, the LSFD method mentioned above was used to estimate the strain mode shape vector according to Equations (7) and (8). All the strain mode shapes here are normalised with respect to the strain amplitude of the point from which the distance is 35 mm to the fixed end in the length direction. The comparisons of the normalised strain mode shapes for the first four modes in the length direction (*Z* direction) obtained by FEA simulation calculation and FBG sensor measurements are shown in Figure 7. It is observed that the normalised strain mode shapes of these two approaches are in good agreement.

**Figure 8:**
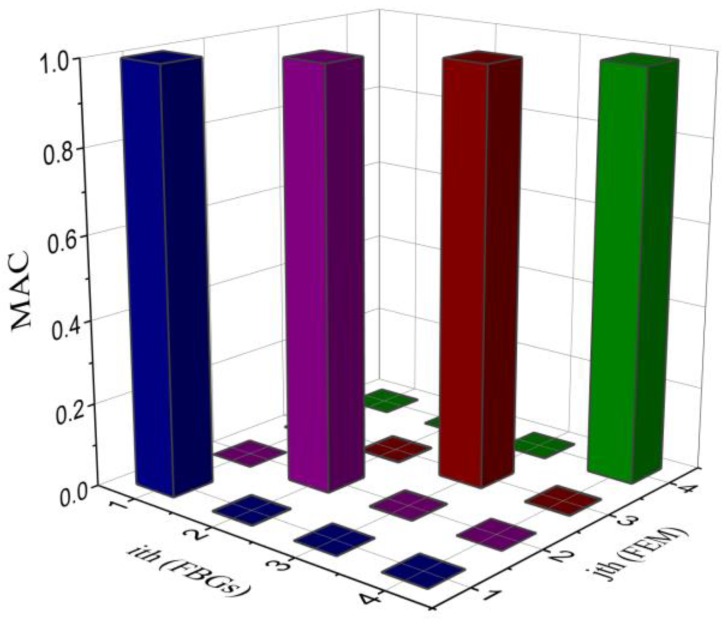
MAC correlation of strain mode shapes between the results of experimental testing and FEA calculation.

Modal assurance criteria (MAC) are usually employed to determine the quality of the measured mode shapes, which ranges between 0 and 1 [23]. If the value of the MAC is close to 0, then there is little correlation between the two modes. However, if the value of the MAC approaches 1.0, it means that the two modes are highly correlated and almost identical. The correlation of the strain mode shapes between experimental testing by FBG sensors and FEA calculation (shown in Figure 7) was calculated using the MAC and the results are shown in Figure 8. The diagonal MAC values are close to 1.0, and the non-diagonal values are nearly close to zero. It is indicated that there is a high correlation between the two strain mode shapes (acquired by experiments using FBG sensors and FEA simulation), and the experimental modal testing results by the FBG sensors are accurate and reliable.

## 5. Application

**Figure 9:**
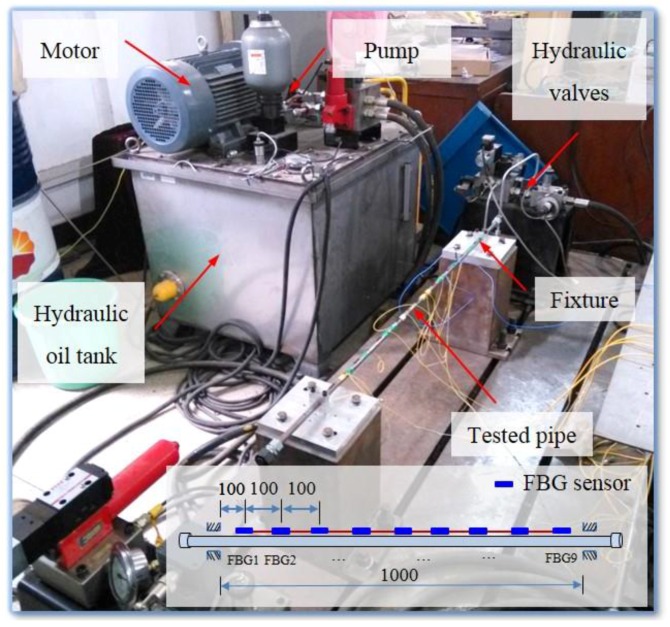
Hydraulic platform and tested pipe with FBG sensors.

The proposed strain modal analysis method using FBG sensors was used to measure the strain modal parameter of hydraulic pipe in the hydraulic system, as shown in Figure 9. The tested pipe was filled with hydraulic oil and nine FBG sensors were adopted to detect its dynamical strain responses on the surface of the pipe under impacting load induced by a hammer. The interval of the FBG sensors is 100 mm and installation positions of the FBG sensors are shown in Figure 9. The hydraulic platform includes a motor, pump, hydraulic oil tank, hydraulic valves, tested hydraulic pipe, and fixtures. The tested hydraulic pipe was fixed by two fixtures at two ends of the pipe.

**Figure 10:**
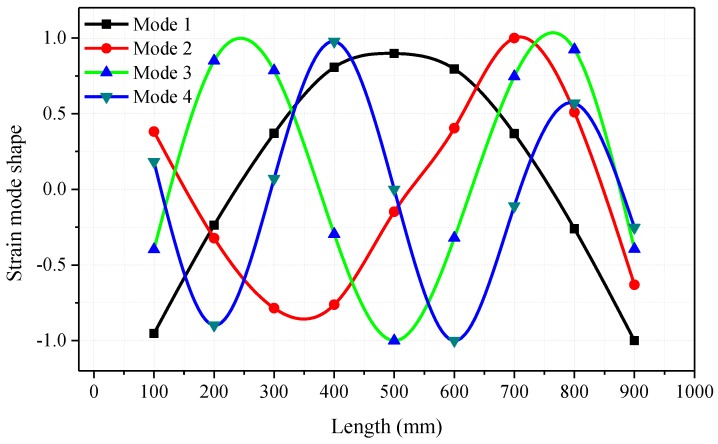
Normalised strain mode shapes along the length direction of the tested pipe.

A set of SFRFs could be obtained from the data acquired by the FBG sensors, and the first four natural frequencies and damping ratios of the tested hydraulic pipe are estimated and shown in Table 3. The first four normalised strain mode shapes along the length direction of the tested pipe are shown in Figure 10. The strain distributions of the tested pipe under different modes could be found in the strain mode shapes.

## 6. Conclusions

As strain measurements are directly related to stress, fatigue and failure, strain modal analysis has a great potential in evaluating the dynamic performance of small and light pipes. With some unique advantages, FBG sensors could be used for the experimental strain modal analysis of small and light pipes to detect distributed dynamic strain responses, where the size and weight of sensing elements are restricted. In this paper, the experimental strain modal analysis of small and light pipes using FBG sensors is investigated. Strain modal analysis of a cantilever small-diameter pipe using FBG sensors has been carried out. The Natural frequencies and strain mode shapes are experimentally acquired and compared with the results of a reference accelerometer and finite element simulation analysis. The good agreement between the results and the MAC values indicate that FBG sensors can be used for the experimental strain modal analysis of small and light pipes. The first four strain modal parameters of the hydraulic pipe were obtained by the proposed strain modal testing method.

## Figures and Tables

**Table 1:** The locations of FBG (Fibre Bragg Grating) sensors.

Sensor Number	Wavelength (nm)	Distance from Fixed End (mm)
FBG1	1295.388	35
FBG2	1290.417	125
FBG3	1288.557	215
FBG4	1309.855	305
FBG5	1304.282	395
FBG6	1298.596	485
FBG7	1309.965	575
FBG8	1288.398	665
FBG9	1290.245	755
FBG10	1304.615	845
FBG11	1298.676	935

**Table 2:** The estimated natural frequencies and damping ratios of the pipe.

Mode Number	Natural Frequency (Hz)	Damping Ratio (%)
1	14.8	0.471
2	92.8	0.204
3	259.0	0.195
4	507.3	0.221

**Table 3:** The natural frequencies and damping ratios of the tested pipe.

Mode Number	Natural Frequency (Hz)	Damping Ratio (%)
1	74.6	0.665
2	209.8	0.305
3	410.4	0.145
4	670.6	0.195
